# Supplementary material for: LEAFY COTYLEDON1 expression in the endosperm enables embryo maturation in Arabidopsis
Source: Nat Commun. 2021 Jun 25;12:3963. doi: 10.1038/s41467-021-24234-1 (PMC8233312; doi:10.1038/s41467-021-24234-1)
Supplement: Supplementary file 1 — Supplemental information [file 41467_2021_24234_MOESM1_ESM.pdf]

LEAFY COTYLEDON1 expression in the endosperm enables embryo maturation in Arabidopsis

Song *et al*.

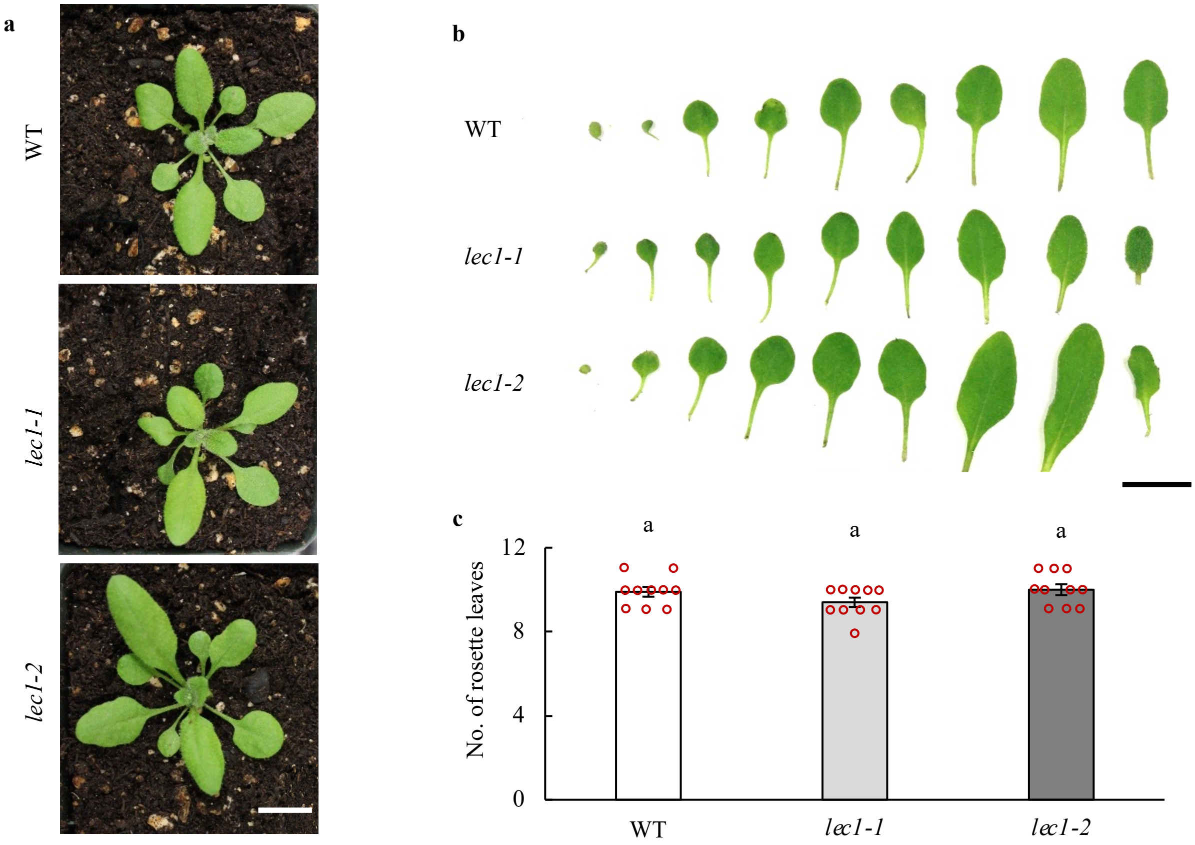

Supplementary Fig. 1. Morphology of embryo-rescued *lec1* homozygous plants. a. Images of wild type (WT, Col-0), *lec1-1*, and *lec1-2* plants at 4 weeks after germination. The *lec1-1* and *lec1-2* plants were grown from rescued embryos. Scale bar: 10 mm. **b.** Exhibition of rosette leaves from one plant of each line. Scale bar: 10 mm. **c.** Number of rosette leaves of Col-0, *lec1-1*, and *lec1-2* plants at bolting stage. Statistical difference analyses was conducted with one-way ANOVA followed by the post-hoc Tukey multiple comparison tests ($p < 0.05$). Values are mean ± standard error of ten biological replicates (one plant for each biological replicate).

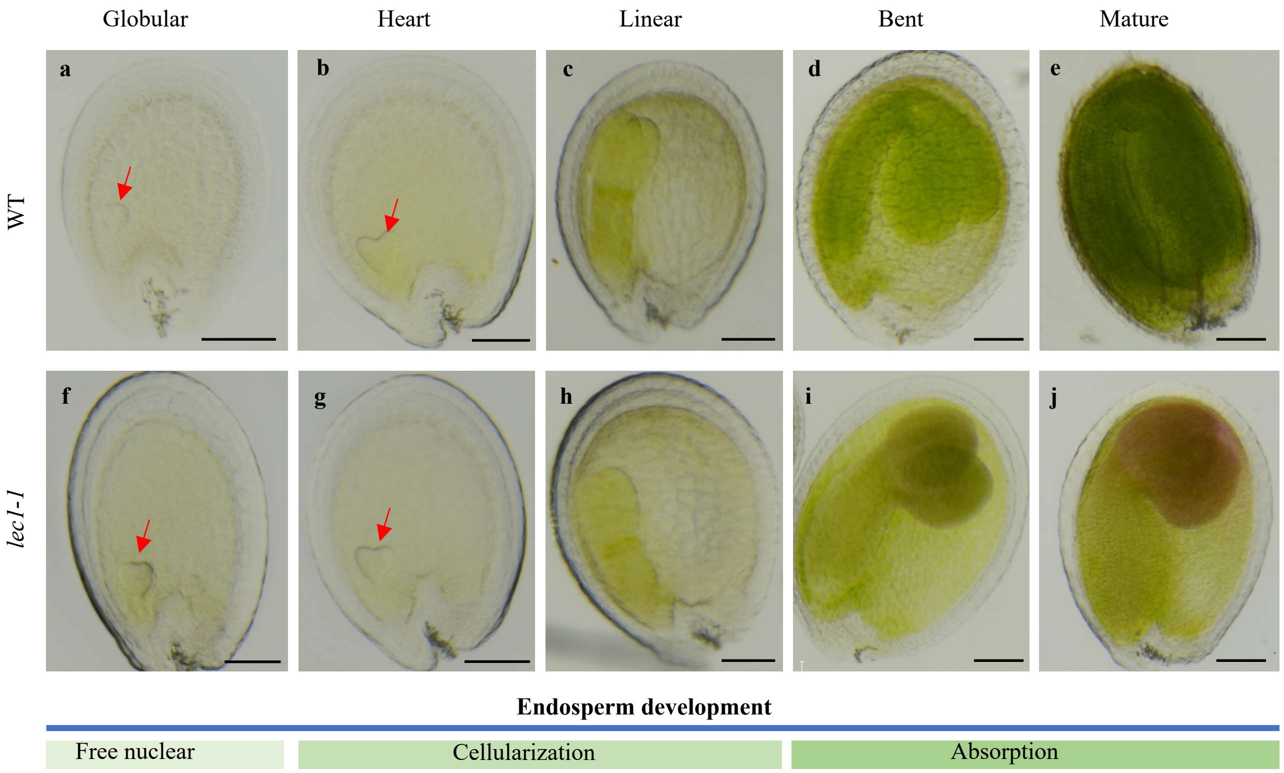

Supplementary Fig. 2. Seed morphology of Col-0 and *lec1-1* at different developmental stages. a-e. WT (Col-0) seed at globular (**a**), heart (**b**), linear (**c**), bent (**d**), and maturation (**e**) stages. **f-j**. *lec1-1* seed at globular (**f**), heart (**g**), linear (**h**), bent (**i**), and maturation (**j**) stages. Red arrows point at the embryos. The corresponding development phases of endosperm are indicated at the bottom. Five seeds at each stage from each genotype were examined in this experiment. This experiment was repeated three times. Scale bar: 100 μm.

a

Self pollination	Genotype of progeny (%)				*n*
	Aborted (fis⁻)	*Aborted (cdka;1⁻)*	*lec1⁻*	*Normal*	
fis2-6$^{+/-}$	50	N.A.	N.A.	50	160
cdka;1$^{+/-}$	N.A.	50	N.A.	50	146
fis2-6$^{+/-}$ *lec1-1*$^{-/-}$	52	N.A.	48	0	153
cdka;1$^{+/-}$ *lec1-1*$^{-/-}$	N.A.	49	51	0	120

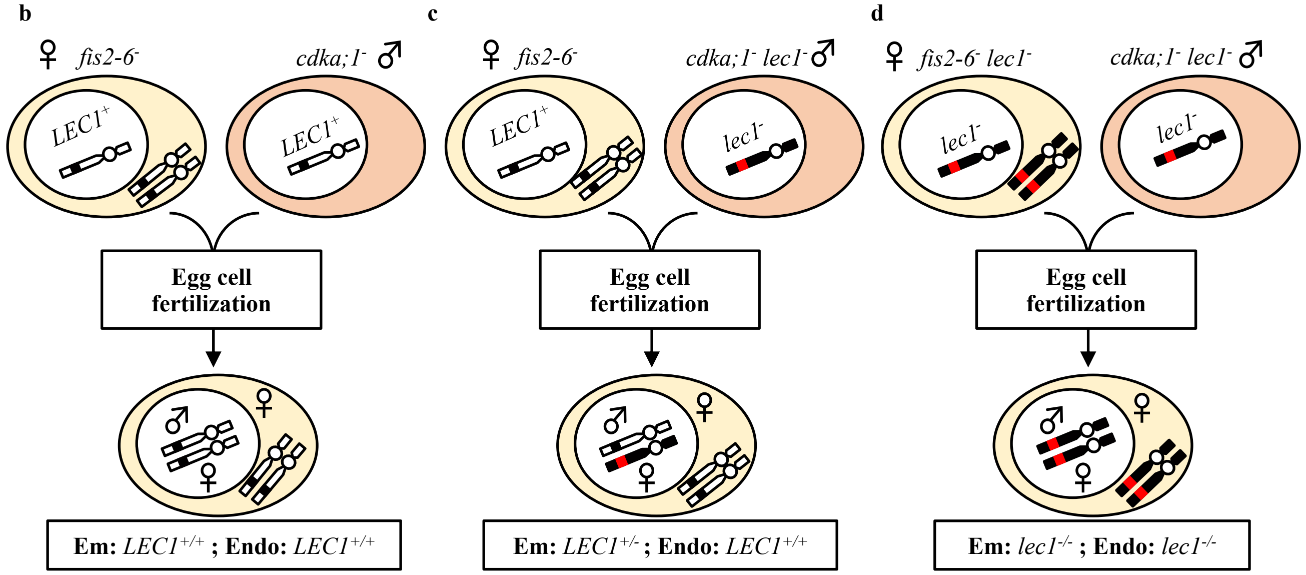

e

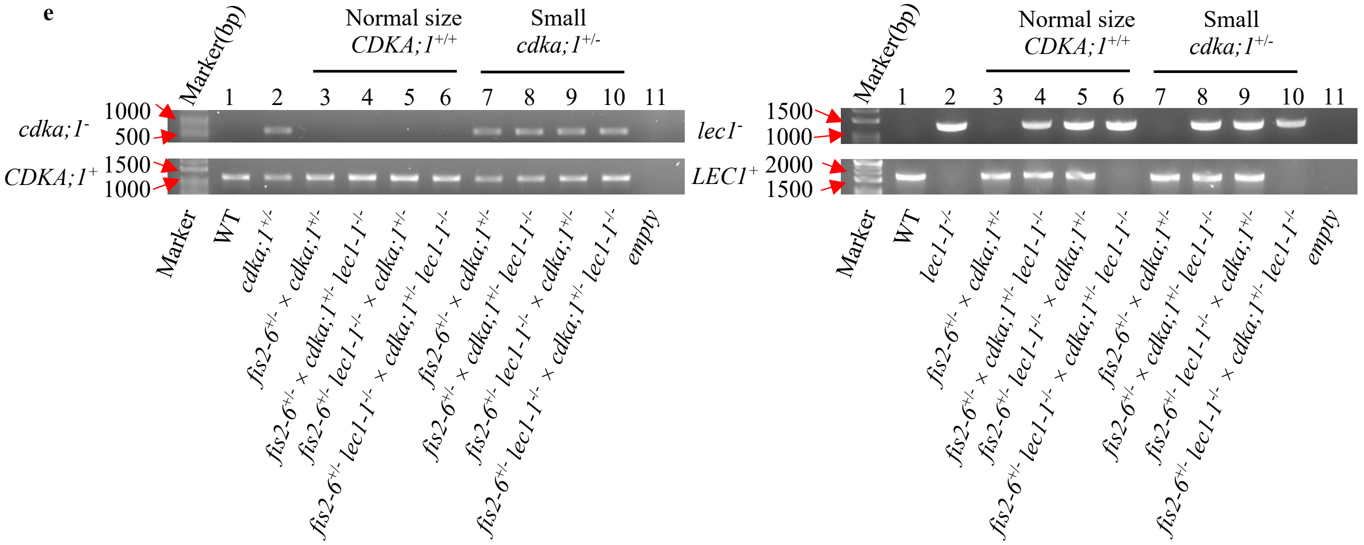

Supplementary Fig. 3. The four mutant lines used in the bypassing test: segregation of their self-crossed progeny seeds and illustration of the control crosses. a. Percentage of various progeny produced from self-pollinations of *fis2-6*$^{+/-}$, *cdka;1*$^{+/-}$, *fis2-6*$^{+/-}$ *lec1-1*$^{-/-}$, and *cdka;1*$^{+/-}$ *lec1-1*$^{-/-}$. n, number of seeds scored. N.A., not applicable. Scale bar: 100μm. **b-d**. Cartoons showing the three genetic crosses used as controls. (**b**) A *fis2-6*$^{-}$ female gamete crossing with *a cdka;1*$^{-}$ pollen to generate a small seed with *LEC1*$^{+/+}$ endosperm and *LEC1*$^{+/+}$ embryo. (**c**) A *fis2-6*$^{-}$ female gamete crossing with *a cdka;1*$^{-}$ *lec1*$^{-}$ pollen to generate a small seed with *LEC1*$^{+/+}$ endosperm and *LEC1*$^{+/-}$ embryo. (**d**) A *fis2-6*$^{-}$ *lec1*$^{-}$ female gamete crossing with *a cdka;1*$^{-}$ *lec1*$^{-}$ pollen to generate a small seed with *lec1*$^{-/-}$ endosperm and *lec1*$^{-/-}$ embryo. Red dot, mutant *lec1*$^{-}$ allele; black dot, wild type *LEC1*$^{+}$ allele. The *cdka;1*$^{-}$ pollen only produces one sperm which only fertilizes the egg cell. **e**. Gel electrophoresis image of PCR-based genotyping of plants derived from the normal size seeds (*CDKA;1*$^{+/+}$) and the small seeds (*cdka;1*$^{+/-}$) produced from the bypassing genetic crosses. *cdka;1*$^{-}$ indicates the T-DNA insertion *cdka;1* allele; *CDKA;1*$^{+}$ indicates the wild type *CDKA;1* allele. *lec1*$^{-}$ represents the T-DNA insertion *lec1* allele; *LEC1*$^{+}$ represents the wild type *LEC1* allele. WT (Col-0), *cdka;1*$^{+/-}$, and *lec1-1*$^{-/-}$ plants were used as control. Three plants of each seed type were examined in this experiment and only one set of data is presented here.

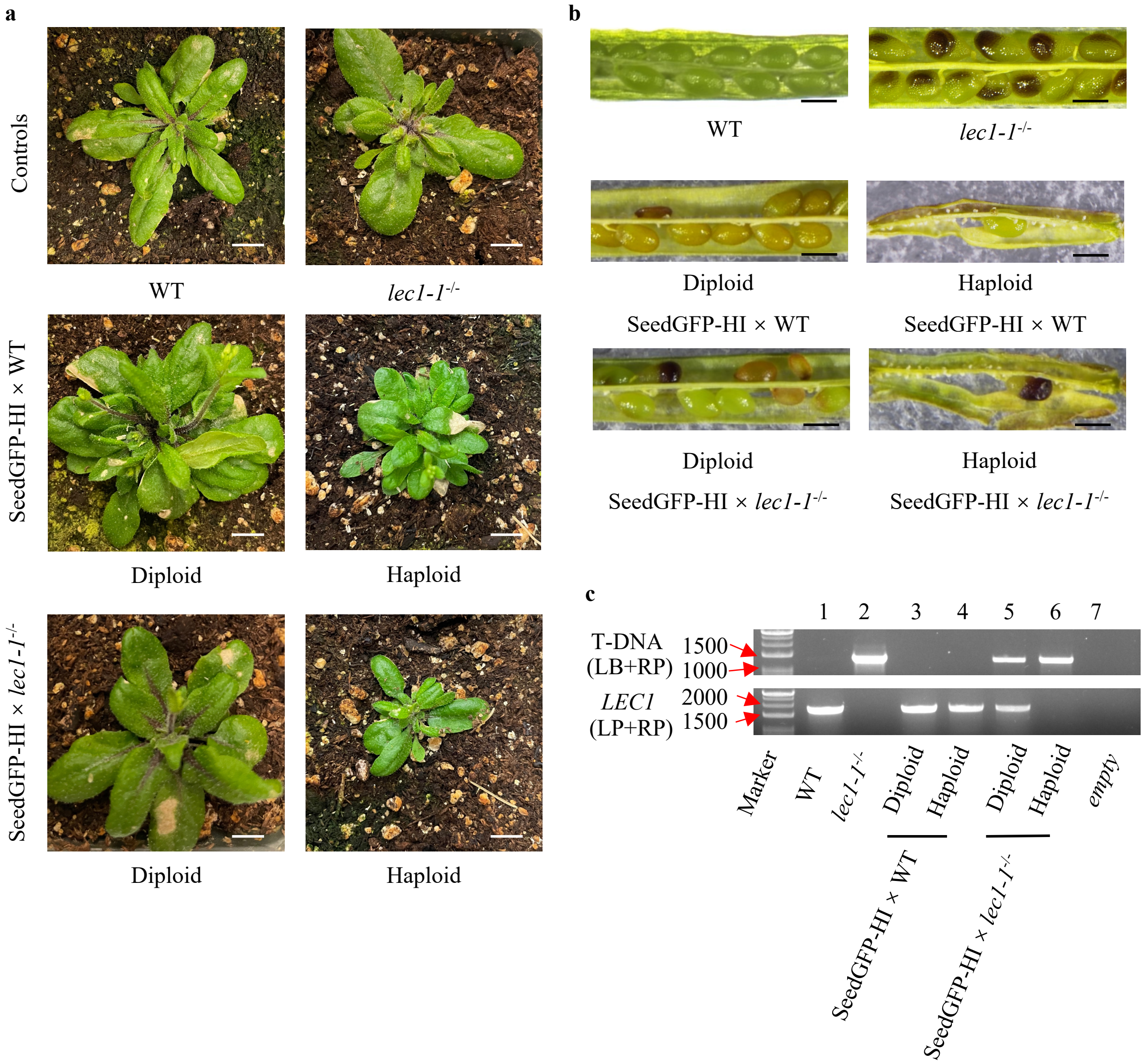

Supplementary Fig. 4. Plant morphology of the diploids and the haploids produced from the SeedGFP-HI crosses. a. Images of 4-week-old plants from the diploid and haploid seeds generated from the SeedGFP-HI × WT and the SeedGFP-HI × *lec1-1*$^{-/-}$ crosses. WT (Col-0) and *lec1-1*$^{-/-}$ plants are shown as controls. Three plants of each genotype were examined. Scale bar: 10 mm. **b**. Representative siliques collected from the diploid and haploid plants. Typical siliques of WT and *lec1-1*$^{-/-}$ are shown as controls. Three siliques from each genotype were examined. Scale bar: 500 μm. **c**. Gel electrophoresis image of PCR-based genotyping of the *lec1* locus in the diploid and haploid plants. LP: Left primer of *LEC1*; RP: Right primer of *LEC1*; LB: Left border primer of T-DNA; WT and *lec1-1*$^{-/-}$ were used as controls. Three plants of each genotype were genotyped and only one set of data was shown here.

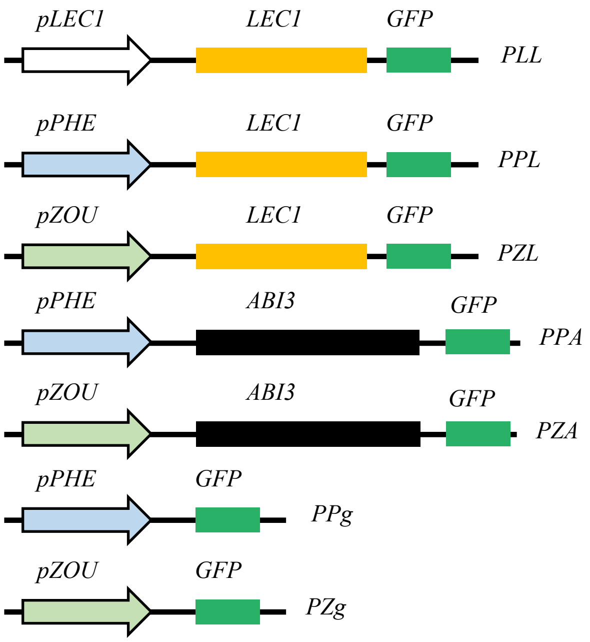

Supplementary Fig. 5. Constructs used for plant transformation. Schematic diagrams showing the transgene structures used for each independent plant transformation.

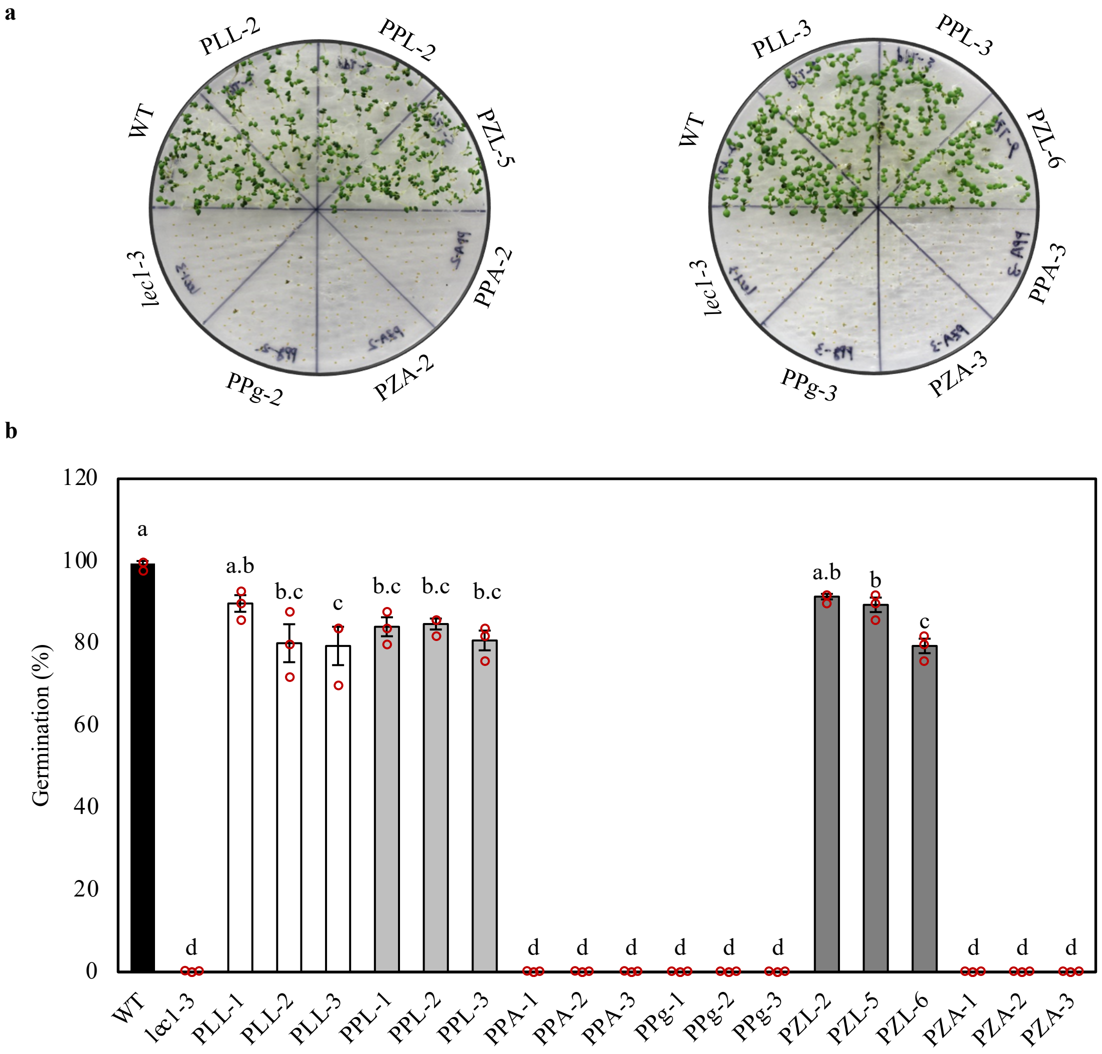

Supplementary Fig. 6. Germination of the transgenic seeds in Ler-0 background. a. Germination of seeds (T2) from two independent lines of the same construct. **b**. Germination rates of T2 seeds (3 independent lines of each transformation). a to d indicate statistical difference with one-way ANOVA followed by the post-hoc Tukey multiple comparison tests ($p < 0.05$). Values are mean ± standard error, based on three biological replicates (50 seeds were used for one biological germination test).

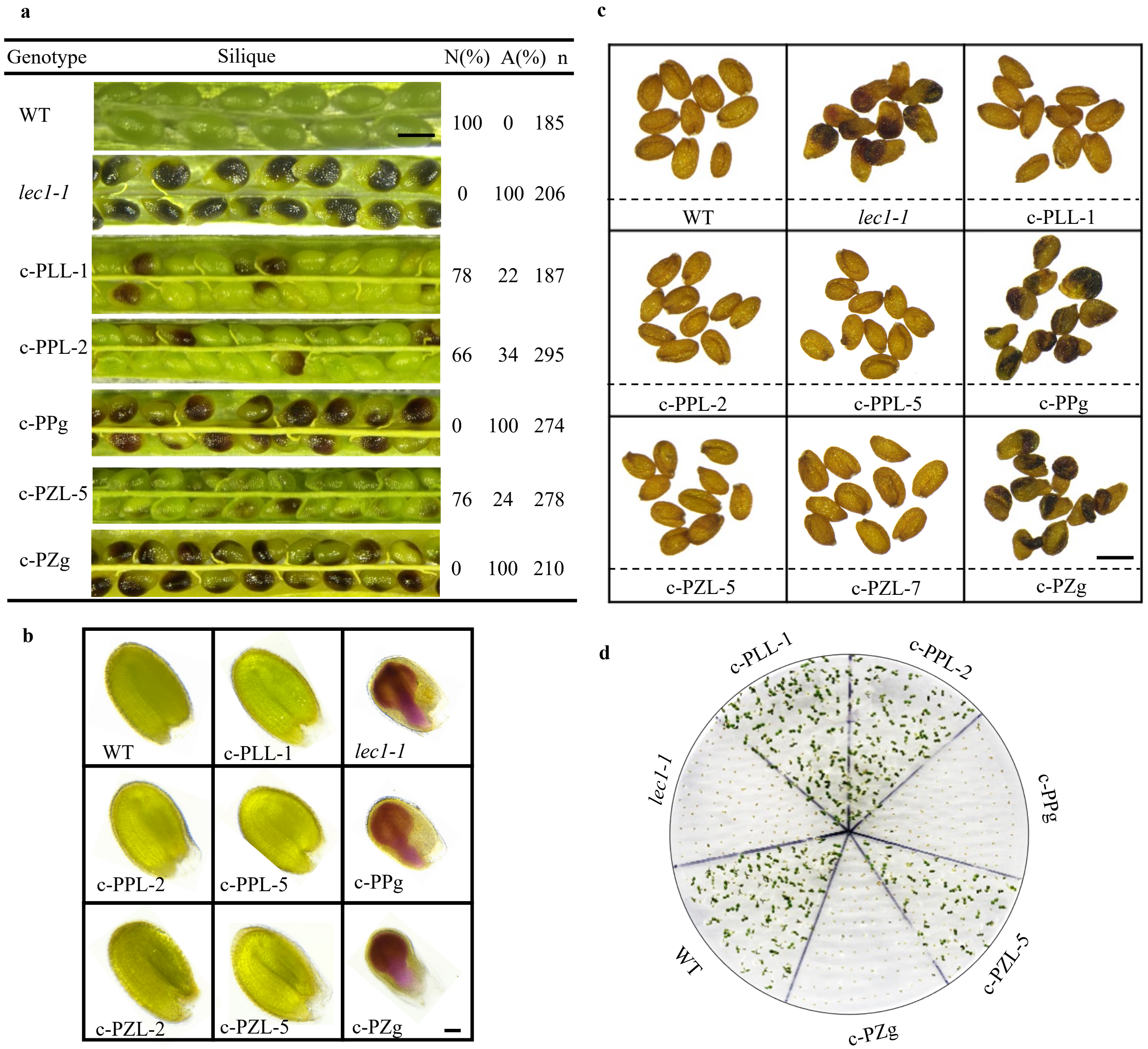

Supplementary Fig. 7. Gain-of-function of *LEC1* in the endosperm rescues *lec1-1* seed phenotype. a. Green mature siliques collected from T1 plants of each of the transgene constructs. A typical silique of T1 hemizygous plants c-PLL-1 (*pLEC1::LEC1-GFP lec1-1*$^{-/-}$), c-PPL-2 (*pPHE::LEC1-GFP lec1-1*$^{-/-}$), c-PZL-2 (*pZOU::LEC1-GFP lec1-1*$^{-/-}$), and c-PZL-5 (*pZOU::LEC1-GFP lec1-1*$^{-/-}$) contain [WT] seeds and [*lec1-1*] seeds. c-PPg (*pPHE::GFP lec1-1*$^{-/-}$), c-PZg (*pZOU::GFP lec1-1*$^{-/-}$) only produce [*lec1-1*] seeds. [WT] indicates WT phenotype, [*lec1-1*] indicates *lec1-1* phenotype. N, normal seeds; A, abnormal seeds (*lec1-1*); n, number of seeds scored. At least 5 independent individual transgenic lines were examined for each construct transformation. Scale bar: 500 μm. **b.** Images of a typical seed embryo from the transgenic lines. Three seeds of each genotype were examined. Scale bar: 100 μm. **c.** Phenotypes of dry seeds from the transgenic lines, including WT (Col-0), *lec1-1*, c-PLL-1, c-PPL-2, c-PPL-5, c-PPg, c-PZL-5, c-PZL-7, and c-PZg. Ten seeds of each genotype were examined. Scale bar: 300 μm. **d.** Germination of seeds with different transgenic backgrounds at day 7. This germination test was repeated three times, 50 seeds were used for one biological germination test.

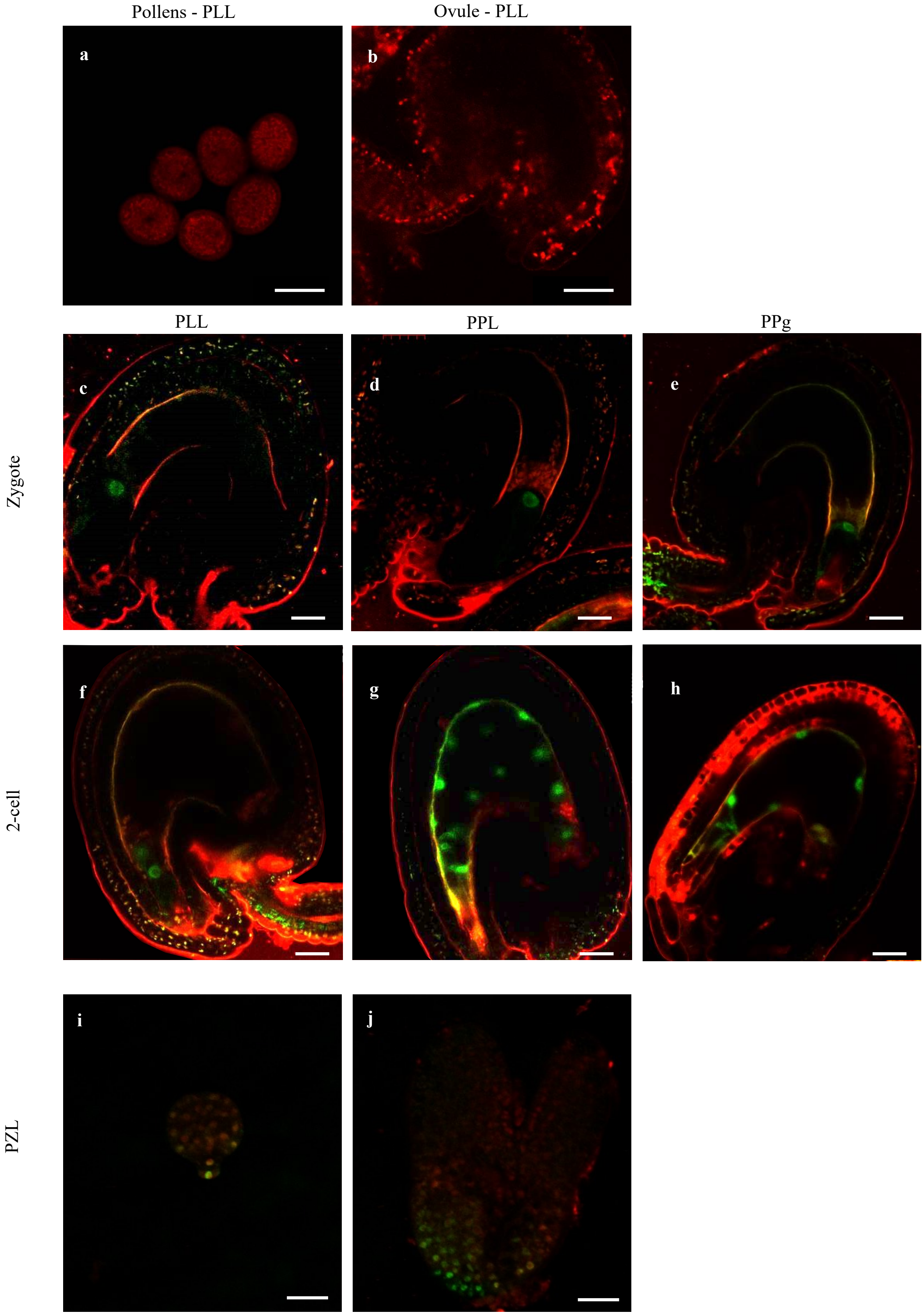

Pollens - PLL
Ovule - PLL
a
b
PLL
PPL
PPg
Zygote
c
d
e
2-cell
f
g
h
PZL
i
j

Supplementary Fig. 8. GFP signals in the transgenic seeds. **a.** No GFP signal was detected in the PLL pollens. This experiment was repeated three times. **b.** No GFP signal was detected in the PLL ovules**.** Five ovules were examined. **c-h.** Original images corresponding to **Fig. 4a-f**. **i-j**. GFPs were detected in the PZL globular (**i**) and heart embryos (**j**). GFP signals are shown in green colour. Cell walls stained with propidium iodide (PI) are shown in red. Shown are merged confocal images from GFP and PI channels. In this experiment, Five seeds or embryos at each stage from each line were examined. Scale bar: 20 μm.

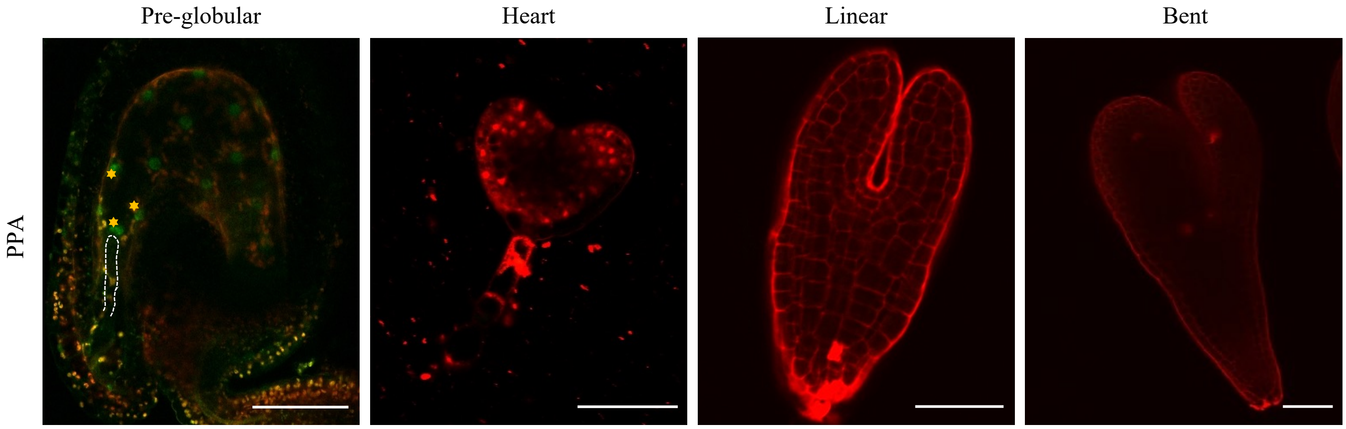

Supplementary Fig. 9. Detection of GFP signals in PPA seeds. Confocal images showing GFP signal positive in the endosperm nuclei at pre-globular stage and GFP negative in the embryos (pre-globular, heart, linear and bent). White dash line outlines embryo; amber stars indicate endosperm nuclei. GFP signals are shown in green colour. Cell walls stained with PI are shown in red. Shown are merged confocal images from GFP and PI channels. Three seeds or embryos at each stage were examined. Scale bar: 50 μm.

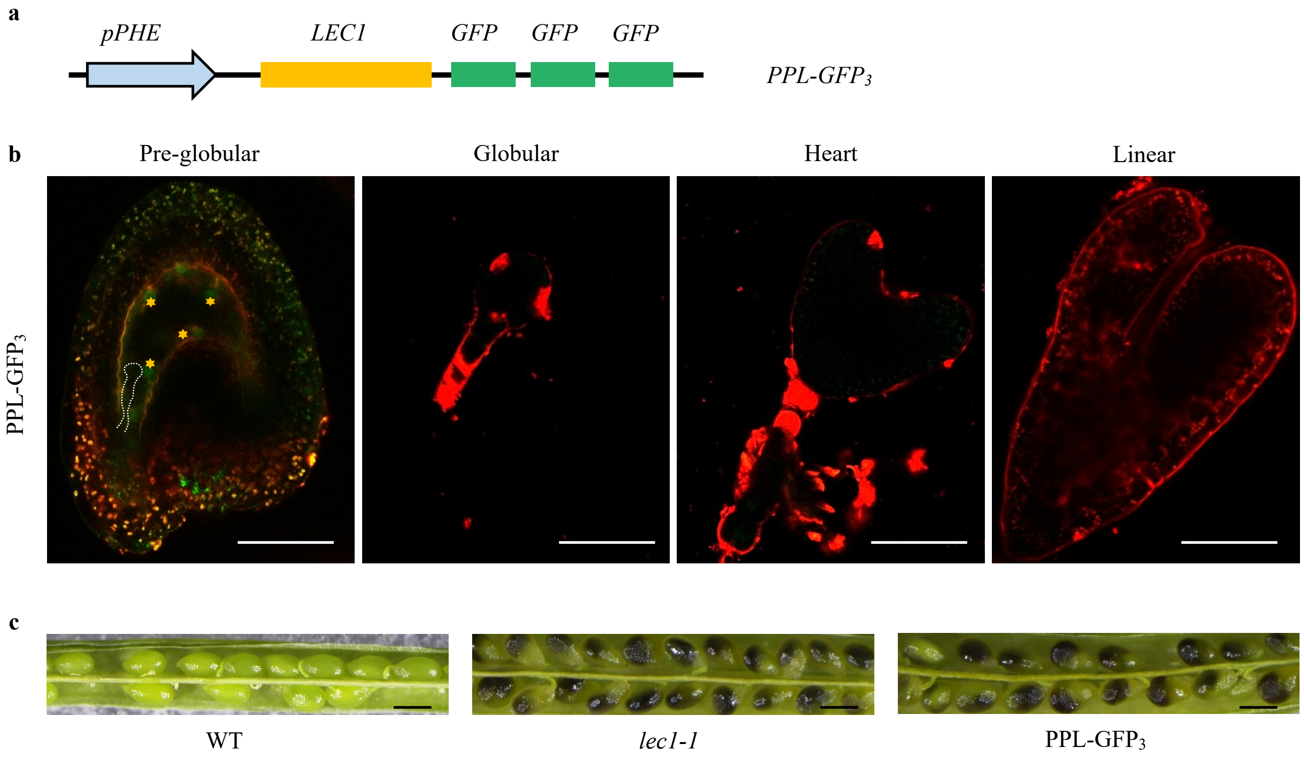

Supplementary Fig. 10. The *PPL-GFP$_3$* transgene failed to rescue *lec1-1* seed phenotype. a. Schematic diagram showing the transgene structure of *PPL-GFP$_3$* (*pPHE1::LEC1-GFP$_3$*) used for transforming *lec1-1* plants. **b**. Confocal images showing GFP signal positive in the endosperm nuclei at pre-globular stage and GFP negative in the embryos (pre-globular, globular, heart, and linear). White dash line outlines embryo; amber stars indicate endosperm nuclei. GFP signals are shown in green colour. Cell walls stained with PI are shown in red. Shown are merged confocal images from GFP and PI channels. Three seeds or embryos at each stage were examined. Scale bar: 50 μm. **c**. Images of representative green mature siliques collected from WT (Col-0), *lec1-1*, and T1 plants of PPL-GFP$_3$ (*pPHE1::LEC1-GFP$_3$ lec1-1$^{-/-}$*). Three independent transgenic lines of PPL-GFP$_3$ were assessed. Scale bar: 500 μm.

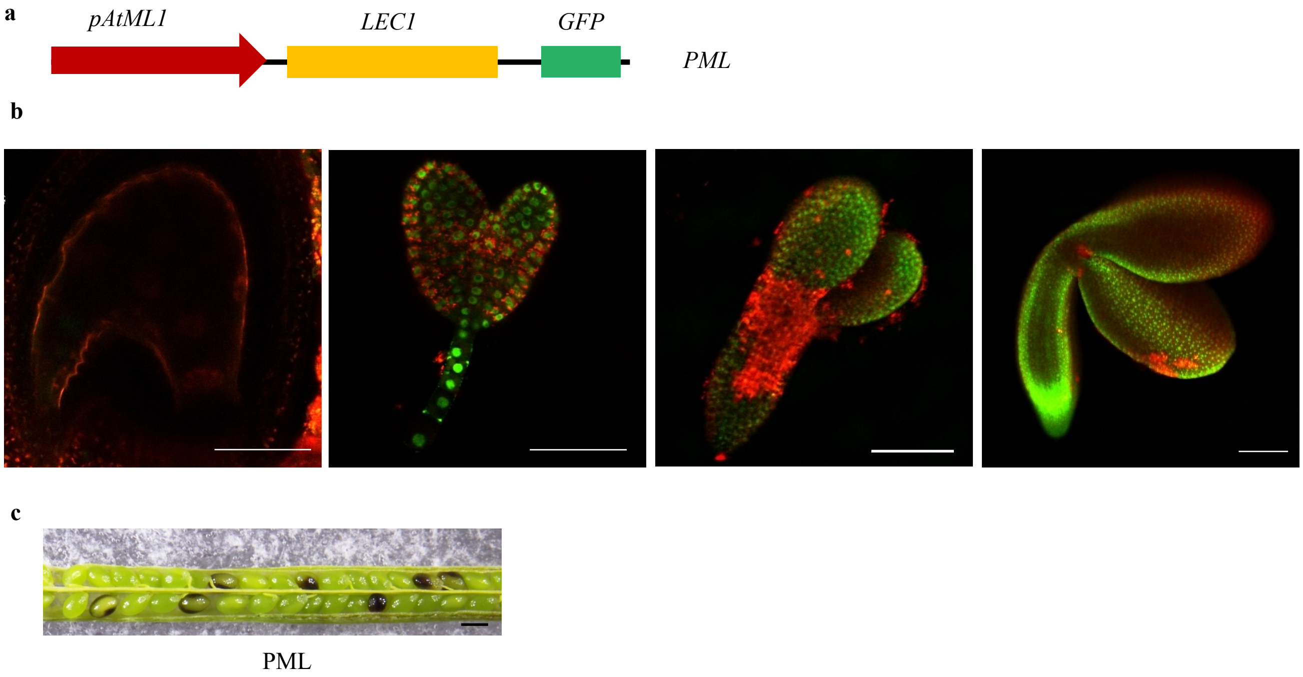

Supplementary Fig. 11. The PML transgene rescued *lec1-1* seed phenotype. a. Schematic diagram showing the transgene structure of *PML* (*pAtML1::LEC1-GFP*) used for transforming *lec1-1* plants. **b**. Confocal images showing GFP signal negative in the endosperm nuclei at pre-globular stage and GFP positive in the embryos (heart, linear, and mature green). GFP signals are shown in green colour. Cell walls stained with PI are shown in red. Images are shown as the merged channel of GFP and PI. Shown are merged confocal images from GFP and PI channels. Three seeds or embryos at each stage were examined. Scale bar: 50 μm. **c**. Image of a representative green mature silique collected from PML (*pAtML1::LEC1-GFP lec1-1*$^{-/-}$) T1 plants. Three independent transgenic lines of PML were assessed. Scale bar: 500 μm.

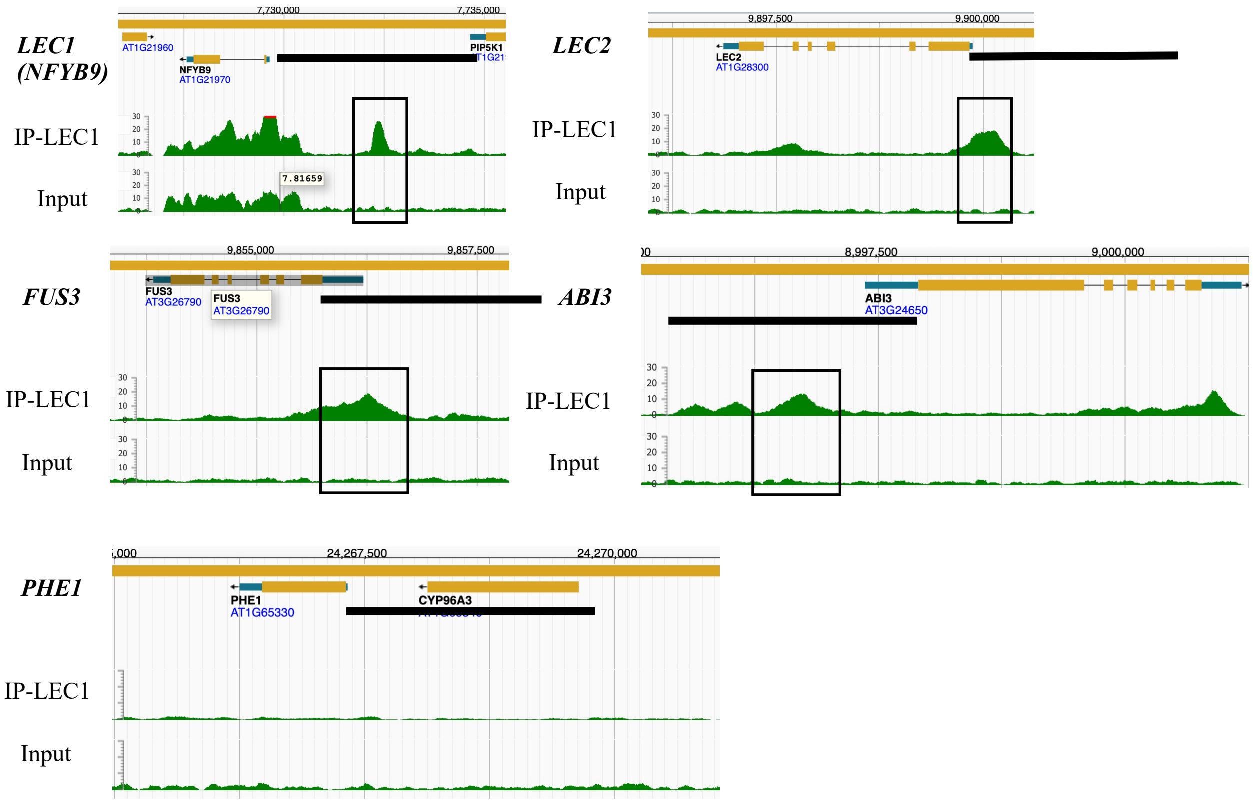

Supplementary Fig. 12. Binding of LEC1 at the promoters of *LEC1*, *LEC2*, *FUS3*, and *ABI3*. Genome browser view of ChIP-seq signals showing the binding activity of LEC1 at the promoter-TSS (transcription start site) regions of *LEC1*, *LEC2*, *FUS3* and *ABI3*. *PHE1* is shown as negative control. Binding signals of LEC1 on the promoter regions of *LEC1*, *LEC2*, *FUS3* and *ABI3* are highlighted with black frames. Black bars indicate promoter regions that are defined as 2kb upstream of TSS. Note: the LEC1 genomic occupancy patterns presented here are based on published LEC1 ChIP-seq data by Pelletier et al. (2017).

Supplementary Table 1. Primers used in this study

Name	Primer sequences (5' - 3')	Target site
LB	ATTTTGCCGATTTCGGAAC	Left border T-DNA primer for *lec1-1*, *cdka:1* (Salk binary vector pROK2)
SALK_131219-LP	ATGGAACGTGGAGCTCCCTTCTC	Left border for genotyping *lec1-1*
SALK_131219-RP	CTTATACTGACCATAATGGTC	Right border for genotyping *lec1-1*
cdka-LP	TCAAACAAGTTTGGTTTTGGC	Left border for genotyping *cdka:1*
cdka-RP	TTCCTTGTTCATATGTTCCCG	Right border for genotyping *cdka:1*
HI-ECORV-FOR	GGTGCGATTTCTCCAGCAGTAAAAATC	Forward primer for dCAPS assay in genotyping HI line
HI-ECORV-REV	CTGAGAAGATGAAGCACCGGCGATAT	Reverse primer for dCAPS assay in genotyping HI line
F-pro-LEC1	GGGTTTAAACTGCAAATGGTAATCATTATGTCTG	Forward primer for promoter *pLEC1* cloning
R-LEC1	TGGCGCGCCACTTATACTGACCATAATGGTC	Reverse primer for genomic DNA *LEC1* cloning
F-LEC1	GGGTTTAAACCCTAGGATGGAACGTGGAGCTCCCTTCTC	Forward primer for genomic DNA *LEC1* cloning
Mlu1-LEC1-R	GGACGCGTCTTATACTGACCATAATGGTC	Reverse primer for *pPHE1::LEC1* cloning
F-pro-PHE1	GGGTTTAAACACTGTTGATCCGGTGAATATCC	Forward primer for promoter pPHE1 cloning
R-pro-PHE1	TCACCTAGGATCTCTTATCTTTTTCTTTTGTGTATTTTG	Reverse primer for promoter *pPHE1* cloning, fusing with *LEC1-GFP*
R-pro-PHE1-1	TGGCGCGCCAATCTCTTATCTTTTTCTTTTGTGTATTTTG	Reverse primer for promoter *pPHE1* cloning, fusing with *GFP* only
F-pro-ZOU	GGGTTTAAACTACCACCCTATACTTATTAGACAG	Forward primer for promoter *pZOU* cloning
R-pro-ZOU	TCACCTAGGATTGAATTGAATGCTCATTTTACC	Reverse primer for promoter *pZOU* cloning, fusing with *LEC1-GFP*
R-pro-ZOU-1	TGGCGCGCCAATTGAATTGAATGCTCATTTTACC	Reverse primer for promoter *pZOU* cloning, fusing with *GFP* only
Pme1-Mlu1-GFP-F	GGGTTTAAACACGCGTTCGAGGGGGGGCCC	Forward primer for *GFP-GFP* cloning
Asc1-GFP-R	TGGCGCGCCAGTGGTGGTGGTGGTG	Reverse primer for *GFP-GFP* cloning
F-ABI3	GGGTTTAAACCCTAGGATGAAAAGCTTGCATGTGGCGG	Forward primer for genomic DNA *ABI3* cloning
R-ABI3	TGGCGCGCCATTTAACAGTTTGAGAAGTTGGTG	Reverse primer for genomic DNA *ABI3* cloning
pAtML1-F	GGGTTTAAACCATTTACACATCCTGTCG	Forward primer for promoter *pAtML1* cloning
pAtML1-R	TCACCTAGGGTGGATTCAGGGAG	Reverse primer for promoter *pAtML1* cloning
LEC2-rtF	CACAGGAGAATTTGTGAAGC	*LEC2* forward primer for quantitative RT-qPCR
LEC2-rtR	CCTCTTCGTCTCTTGGTATG	*LEC2* reverse primer for quantitative RT-qPCR

Supplementary Table 1. continued

Name	Primer sequences (5' - 3')	Target site
ABI3-rtF	GTGATGGAGACTCAGTTACC	*ABI3* forward primer for quantitative RT-qPCR
ABI3-rtR	TTTTGGCAAAACGATCCTTC	*ABI3* reverse primer for quantitative RT-qPCR
FUS3-rtF	ATGATACTCCCGAAGAAAGC	*FUS3* forward primer for quantitative RT-qPCR
FUS3-rtR	TGTGTTTTCTAGCACGTACA	*FUS3* reverse primer for quantitative RT-qPCR
CACS-rtF	ACTCAGGAAGGTGTACGGTCA	*CACS* forward primer for quantitative RT-qPCR
CACS-rtR	TGCATTTGGAACAGGTTTGT	*CACS* reverse primer for quantitative RT-qPCR
GFP-rtF	GGAGAAGAACTTTTCACTGG	*GFP* forward primer for quantitative RT-qPCR
GFP-rtR	CCTTCACCCTCTCCACTGAC	*GFP* reverse primer for quantitative RT-qPCR
