# Supplementary material for: LEAFY COTYLEDON1 expression in the endosperm enables embryo maturation in Arabidopsis
Source: Nat Commun. 2021 Jun 25;12:3963. doi: 10.1038/s41467-021-24234-1 (PMC8233312; doi:10.1038/s41467-021-24234-1)
Supplement: Supplementary file 3 — Reporting Summary [file 41467_2021_24234_MOESM3_ESM.pdf]

nature research

Corresponding author(s): Yuhai Cui
Jitao Zou
Jingpu Song

Last updated by author(s): May 30, 2021

Reporting Summary

Nature Research wishes to improve the reproducibility of the work that we publish. This form provides structure for consistency and transparency in reporting. For further information on Nature Research policies, see our Editorial Policies and the Editorial Policy Checklist.

Statistics

For all statistical analyses, confirm that the following items are present in the figure legend, table legend, main text, or Methods section.

n/a	Confirmed	
☐	☒	The exact sample size (*n*) for each experimental group/condition, given as a discrete number and unit of measurement
☐	☒	A statement on whether measurements were taken from distinct samples or whether the same sample was measured repeatedly
☐	☒	The statistical test(s) used AND whether they are one- or two-sided<br>*Only common tests should be described solely by name; describe more complex techniques in the Methods section.*
☒	☐	A description of all covariates tested
☐	☒	A description of any assumptions or corrections, such as tests of normality and adjustment for multiple comparisons
☐	☒	A full description of the statistical parameters including central tendency (e.g. means) or other basic estimates (e.g. regression coefficient) AND variation (e.g. standard deviation) or associated estimates of uncertainty (e.g. confidence intervals)
☐	☒	For null hypothesis testing, the test statistic (e.g. *F*, *t*, *r*) with confidence intervals, effect sizes, degrees of freedom and *P* value noted<br>*Give P values as exact values whenever suitable.*
☒	☐	For Bayesian analysis, information on the choice of priors and Markov chain Monte Carlo settings
☒	☐	For hierarchical and complex designs, identification of the appropriate level for tests and full reporting of outcomes
☒	☐	Estimates of effect sizes (e.g. Cohen's *d*, Pearson's *r*), indicating how they were calculated

Our web collection on statistics for biologists contains articles on many of the points above.

Software and code

Policy information about availability of computer code

Data collection	Confocal images were analyzed with the OLYMPUS FLUOVIEW (Ver.4.2.) software. RT-qPCR data were collected using the Bio-Rad CFX Manager (Ver 3.1)
Data analysis	R v.3.6.3 (http://www.r-project.org/) was used for the statistical analyses. Bartlett tests were performed to verify the equality of the variance across the samples. One-way ANNOVA analyses and post-hoc Tukey tests were conducted to determine the significant difference. Bar graphs were generated by using Microsoft Excel 2016. Bar graphs were generated by using Microsoft Excel 2016.

For manuscripts utilizing custom algorithms or software that are central to the research but not yet described in published literature, software must be made available to editors and reviewers. We strongly encourage code deposition in a community repository (e.g. GitHub). See the Nature Research guidelines for submitting code & software for further information.

Data

Policy information about availability of data

All manuscripts must include a data availability statement. This statement should provide the following information, where applicable:

- Accession codes, unique identifiers, or web links for publicly available datasets
- A list of figures that have associated raw data
- A description of any restrictions on data availability

All lines used in the study will be provided upon signature of appropriate material transfer agreement. All data are available in the main text or the supplementary materials. Source data are provided with this paper.

Field-specific reporting

Please select the one below that is the best fit for your research. If you are not sure, read the appropriate sections before making your selection.

☒ Life sciences ☐ Behavioural & social sciences ☐ Ecological, evolutionary & environmental sciences

For a reference copy of the document with all sections, see nature.com/documents/nr-reporting-summary-flat.pdf

Life sciences study design

All studies must disclose on these points even when the disclosure is negative.

Sample size	Sample sizes were estimated based on our experiences in performing similar experiments and published works. For RT-qPCR, usually three biological replicates were performed in majority published papers. Therefore, we performed three independent biological replicates for each genotype. The exact sample size for each experiment was described clearly in the figure legends.
Data exclusions	No data were excluded from the analysis.
Replication	All attempts at replication were successful.
Randomization	All samples were allocated randomly into experimental groups. All the biological replicates were grown and collected independently at different times, although all the replicates were extracted and processed at the same time.
Blinding	For biological replicates, independent researchers (not related to this project)were asked to collect plant samples, perform experiments, and conduct data analysis.

Reporting for specific materials, systems and methods

We require information from authors about some types of materials, experimental systems and methods used in many studies. Here, indicate whether each material, system or method listed is relevant to your study. If you are not sure if a list item applies to your research, read the appropriate section before selecting a response.

Materials & experimental systems

n/a	Involved in the study	
☒	☐	Antibodies
☒	☐	Eukaryotic cell lines
☒	☐	Palaeontology and archaeology
☒	☐	Animals and other organisms
☒	☐	Human research participants
☒	☐	Clinical data
☒	☐	Dual use research of concern

Methods

n/a	Involved in the study	
☒	☐	ChIP-seq
☒	☐	Flow cytometry
☒	☐	MRI-based neuroimaging
